# Voltage-tunable dual-layer terahertz metamaterials

**DOI:** 10.1038/micronano.2016.25

**Published:** 2016-07-04

**Authors:** Xiaoguang Zhao, Kebin Fan, Jingdi Zhang, George R Keiser, Guangwu Duan, Richard D Averitt, Xin Zhang

**Affiliations:** 1 Department of Mechanical Engineering, Boston University, Boston, MA 02215, USA; 2 Department of Physics, University of California, San Diego, La Jolla, CA 92093, USA; 3 Department of Physics, Boston University, Boston, MA 02215, USA

**Keywords:** broadside-coupled split-ring resonators (BC-SSRs), comb-drive actuator, microelectromechanical systems (MEMS), tunable metamaterials

## Abstract

This paper presents the design, fabrication, and characterization of a real-time voltage-tunable terahertz metamaterial based on microelectromechanical systems and broadside-coupled split-ring resonators. In our metamaterial, the magnetic and electric interactions between the coupled resonators are modulated by a comb-drive actuator, which provides continuous lateral shifting between the coupled resonators by up to 20 μm. For these strongly coupled split-ring resonators, both a symmetric mode and an anti-symmetric mode are observed. With increasing lateral shift, the electromagnetic interactions between the split-ring resonators weaken, resulting in frequency shifting of the resonant modes. Over the entire lateral shift range, the symmetric mode blueshifts by ~60 GHz, and the anti-symmetric mode redshifts by ~50 GHz. The amplitude of the transmission at 1.03 THz is modulated by 74%; moreover, a 180° phase shift is achieved at 1.08 THz. Our tunable metamaterial device has myriad potential applications, including terahertz spatial light modulation, phase modulation, and chemical sensing. Furthermore, the scheme that we have implemented can be scaled to operate at other frequencies, thereby enabling a wide range of distinct applications.

## Introduction

Metamaterials are artificial materials that have been engineered with subwavelength inclusions to have extraordinary electromagnetic (EM) properties, including effective permittivity and permeability^1^. Metamaterials can manipulate EM waves, thereby enabling properties and applications such as negative refractive indices^2^, cloaking^3^, super-lensing^4^, and perfect absorption^5^. As the field of metamaterials has matured over the years, research has focused on realizing functional devices for various applications, including detection^6^, imaging^7^, and chemical sensing^8^, in which exotic EM responses are needed along with the ability to tune metamaterial properties in real-time^9^.

The active tuning of metamaterials can improve functionality by broadening the operating frequency, modulating the magnitude and phase, or controlling the polarization or directionality of an incident beam. Over the past several years, optical^10–13^, thermal^14^, electrical^15–19^, and mechanical^20^ schemes have been utilized to modulate the response of the metamaterials by dynamically altering the properties of materials within the unit cell or by changing the near-field coupling between neighboring resonators^21,22^. Broadside coupled split-ring resonators (SRRs) have been analyzed theoretically and experimentally to evaluate the effect of near field coupling on the response characteristics^23^. On the basis of the understanding of near-field coupling, a tunable metamaterial composed of laterally shifted broadside-coupled split-ring resonators (BC-SRRs) was investigated at the microwave frequencies^24^ and subsequently extended to the terahertz (THz) range^25^. However, in these studies, the BC-SRRs had a fixed lateral shift and could not be dynamically modulated. Recently, a few efforts have been made to shift the frequency response. For example, a post-fabrication tuning scheme was demonstrated to modify the response of multilayer metamaterials^26^. Given that the tuning scheme was based on the thermal deformation of glue used to bond the two layers of SRRs, it was difficult to provide accurate and dynamic control of the metamaterial response.

Microelectromechanical system (MEMS) electrostatic actuators have been used in numerous industrial and personal portable devices for decades because they provide precise, fast, and active control of micro-scale displacements. The marriage of MEMS actuators and metamaterials leads to a plurality of mechanically reconfigurable metamaterials working at the THz and infrared frequencies^27–33^. Of note are comb-drive actuators that can exert large in-plane displacements and are used to drive lateral shift and tune the response of the metamaterials^28–31^.

Single-layer comb-drive metamaterials have been demonstrated by creating a unit cell consisting of a pair of asymmetric SRRs in the same plane, with one fixed to the silicon substrate and the other located on a movable frame^31^. The movable frame is driven laterally by a comb-drive actuator to adjust the relative position between the SRRs in one unit cell, thus enabling a reconfigurable metamaterial response. This scheme can be adopted to build a real-time tunable metamaterial device based on BC-SRRs by stacking two layers of SRRs, the feasibility of which was assessed in our previous work^34^. In this paper, we present a comprehensive study on the dynamic tuning of comb-drive actuated broadside-coupled SRRs.

## Materials and methods

**Figure 1:**
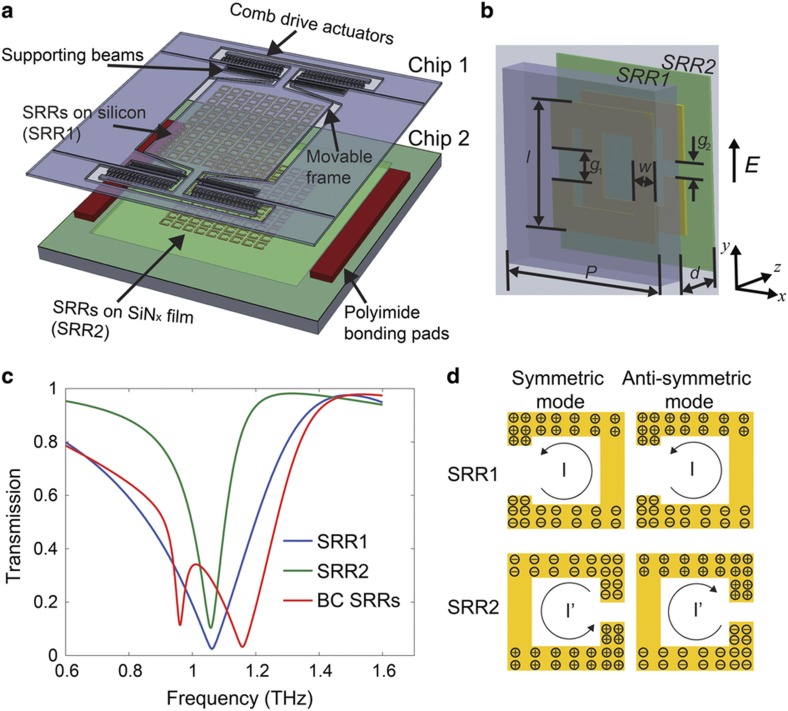
(**a**) Schematic of the exploded view of the tunable metamaterials. (**b**) One unit cell of the tunable metamaterial, including BC-SRRs, where SRR1 is on a silicon frame and SRR2 on a SiN_x_ thin-film. The SRRs are under electric excitation in which the electric field is perpendicular to the gaps of the SRRs. (**c**) Simulated spectra of the individual uncoupled SRRs and broadside-coupled SRRs when *d*=20 μm. (**d**) The surface charge distribution of the symmetric mode and anti-symmetric mode of the BC-SRRs. BC-SSRs, broadside-coupled SRRs; SRRs, split-ring resonators.

As shown in Figure 1a, the tunable metamaterial consists of two arrays of SSRs stacked in a broadside-coupled manner, in which the unit cell is a pair of SRRs separated by an out-of-plane gap and rotated by 180° with respect to each other (Figure 1b). One of the arrays (SRR1) is on a movable silicon frame that can be actuated by a comb-drive actuator, and the other (SRR2) is fixed on a SiN_x_ thin-film. The two arrays are bonded together using a polyimide spacer and separated by an air gap along the *z* axis. The lateral shift between the arrays along the *y* axis (vertical lateral shift: Δ) can be controlled by the comb-drive actuator with an applied voltage. For the demonstration of concept, we designed the SRRs with resonance responses between 0.9 and 1.3 THz. The dimensions of the resonators have been determined from numerical simulations to ensure the working frequency. The side length (*l*) is 40 μm, periodicity (*P*) is 58 μm and line width (*w*) is 11 μm. The gap *g*_1_ is set to 16 μm for SRR1, and *g*_2_ is set to 2 μm for SRR2. Owing to the trade-off in mechanical strength and THz transparency, we have chosen a 10-μm-thick silicon frame as the substrate of SRR1 and a 0.4-μm-thick SiN_x_ film as the substrate for SRR2. The individual SRRs can be considered capacitively loaded subwavelength loops with a LC resonant frequency in which a minimum is achieved in the transmission response^35^. Different gap sizes are used for the two SRRs to compensate for the high permittivity of the thick silicon substrate under SRR1 to match the resonant frequency of the two uncoupled resonators. Given the designed dimensions, the uncoupled individual SRRs resonate at the same frequency of approximately 1.0 THz, as verified by full-wave EM simulations with the electric field parallel to the bars with the gaps, as shown by the blue and green curves in Figure 1c, which plot the transmission as a function of frequency. In the simulations, the dielectric response of the substrates are considered. For the coupled SRRs with an out-of-plane separation distance (*d*) of 20 μm, the simulation reveals two resonances in the transmission spectrum that arise from the coupling between the SRRs, as discussed below (red curve in Figure 1c). The coupled resonance modes are approximately 1.0 THz per the initial design goal. The dimensions of the SRRs can be scaled if the device is desired to operate over a different frequency band.

We can use an equivalent circuit model with mutual inductance and mutual capacitance, accounting for the coupling between the broadside-coupled ring resonators, to describe the behavior of BC-SRRs. For a single SRR, the fundamental mode is the LC resonant mode^35^. In BC-SRRs, magnetic and electric interactions between the resonators should be considered^25^. The magnetic interaction can be modeled as a mutual inductance, and the electric interaction can be modeled as a mutual capacitance. When the interactions between the resonators are sufficiently strong, mode splitting occurs, leading to two resonant frequencies^24^. For the lower-frequency symmetric mode, the currents in the two resonators circulate in the same direction. There is also a higher frequency anti-symmetric mode, in which the currents in the two rings circulate in opposite directions, as shown in Figure 1d. By modifying the interactions between the coupled rings, the frequency difference of the mode splitting can be altered, resulting in a tunable metamaterial. In our structure, tuning is achieved by controlling the lateral distance between the two SRR layers with a comb-drive actuator. The tuning mechanism is discussed in more detail in the following section.

**Figure 2:**
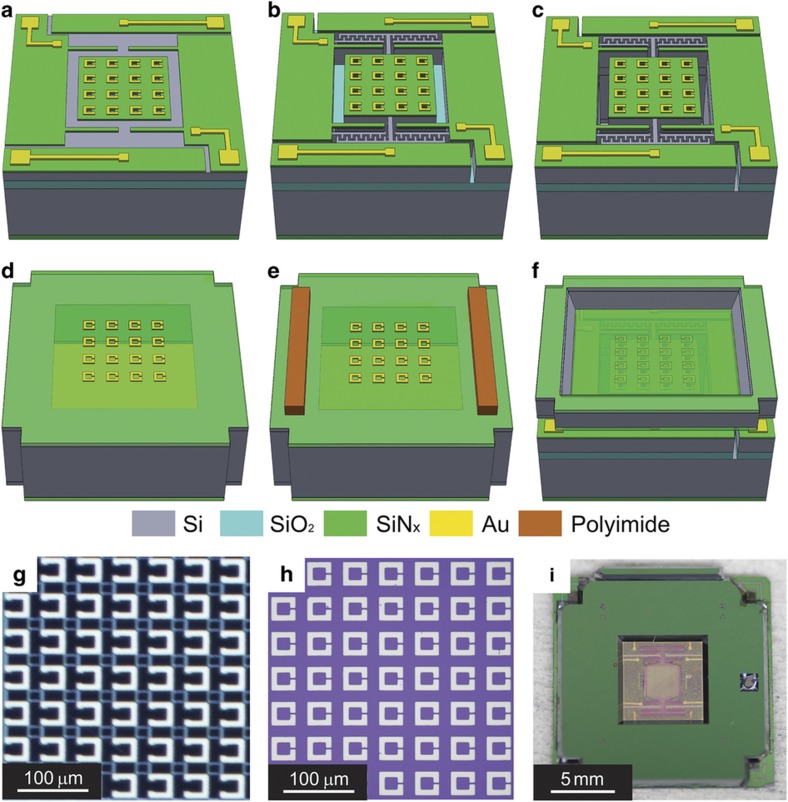
(**a**–**c**) Process flow to fabricate Chip 1 on SOI wafer. (**d**–**e**) Process flow to fabricate chip 2 on silicon wafer. (**f**) Flip-chip bonding to align and bond the two chips. (**g** and **h**) Microscopic images of the SRRs on Chip 1 and Chip 2, respectively. The capacitive gaps are 16 μm in Chip 1 and 2 μm in Chip 2. (**i**) Picture of the bonded device.

The tunable metamaterial is fabricated with bulk micromachining, as shown in Figures 2a–f, which includes two wafer processes and flip-chip bonding^34^. The first wafer is a silicon-on-insulator (SOI) wafer that is coated by low stress SiN_x_ films on both sides. The front side SiN_x_ is etched, and gold electrodes and SRRs are patterned using a lift-off process. Then, we employ deep reactive ion etching (DRIE) to define the comb-drive actuator, movable frame, and supporting beams. Subsequently, the handle wafer is etched thoroughly under the SRR area to eliminate the etalons from multiple reflections of THz radiation in the thick silicon substrate. The movable structures are released by removing the underlying buried oxide layer. For the second wafer, which is a silicon wafer with a double-sided SiN_x_ coating, SRRs are patterned on the front side and the substrate is etched through using KOH anisotropic wet etching from the backside. Then, the polyimide bonding pads are patterned. For simplicity, we name the chips from the SOI wafer as Chip 1 and those from the silicon wafer as Chip 2. The two chips are subsequently integrated using a flip-chip bonder (FC-150, Suss MicroTec AG, Garching bei München, Germany). The detailed process flow is described in the Supplementary Information.

The arrays of SRRs on Chips 1 and 2 are shown in Figures 2g and h. The side width of each unit cell is 40 μm, and the periodicity is 58 μm. This geometry requires at least a lateral displacement of 20 μm to achieve the maximal tuning range to shift the SRRs from a completely aligned to misaligned configuration. The flip-chip-bonded devices (Figure 2i) are wire bonded to a printed circuit board for characterization. The size of the BC-SRRs array is 2 mm by 2 mm.

In fabricating each individual chip, the process parameters are well controlled, and the variations are within ±0.2 μm, which have negligible effects on the response of the metamaterial and the comb-drive actuator. The major source of errors and fabrication challenge is the potential misalignment during the flip-chip bonding, which is ~5 μm. According to our simulations, a 5 μm alignment error would shift the resonant frequency but would have a negligible effect on the tunability.

## Results

**Figure 3:**
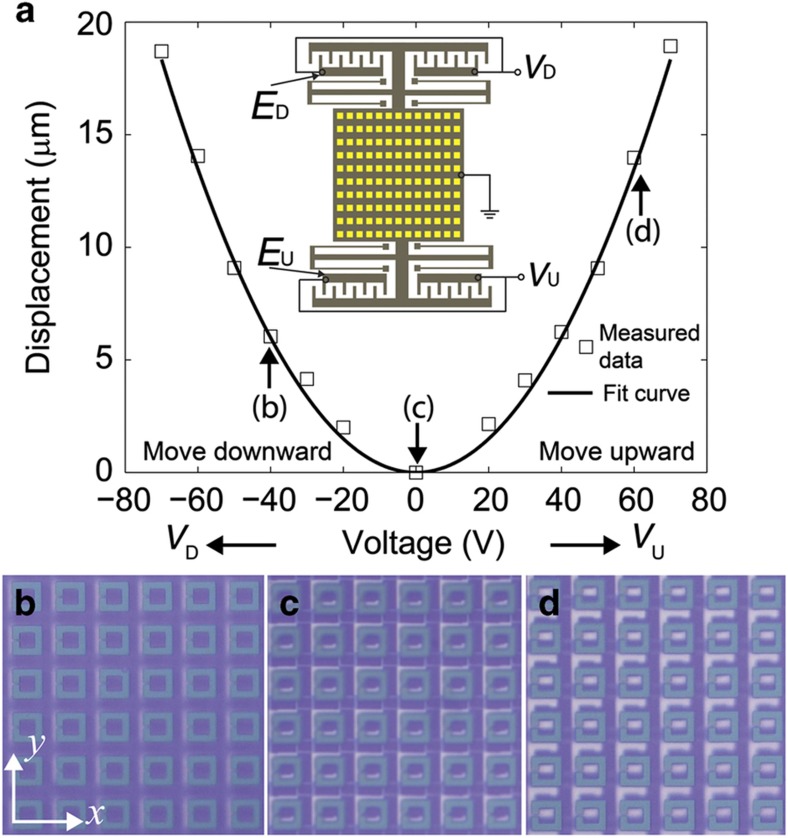
Mechanical characterization of the comb-drive actuator. (**a**) Measured displacement as a function of voltage. The inset shows the electrode configuration of the actuator. (**b**, **c**, and **d**) Microscopic images of the SRRs when *V*_D_=−40 V/*V*_U_=0 V, *V*_D_=*V*_U_=0 V, and *V*_D_=0 V/*V*_U_=60 V, respectively, from which we can see that the SRRs are well aligned in the *x* direction and the lateral shift along the *y* direction can be controlled by the applied voltage. SRR, split-ring resonators.

Mechanical characterization is performed following device integration. A direct current (DC) voltage is applied across one set of comb-drive actuators, and a microscope is utilized to measure the displacement. In the characterization, the movable square frame is grounded while the voltage is applied to the fixed electrodes. We define the electrode that can pull the plate upward as *E*_U_ and the voltage across *E*_U_ as *V*_U_; *E*_D_ (electrode) and *V*_D_ (voltage) can pull the plate downward. The vertical lateral displacement (Δ*y*) exhibits a quadratic dependence on the applied voltage (*V*) as shown in Figure 3a, which can be expressed as Δ*y*=*AV*^2^. The actuation coefficient (*A*) extracted from fitting the measured data is 3.6 nm V^−2^. When no voltage is applied, there is an initial misalignment between the rings in the *y* direction, as shown in Figure 3c. When *V*_D_=40 V and *V*_U_=0 V, the SRRs are aligned (Figure 3b) and Δ=0 μm; when *V*_D_=0 V and *V*_U_=60 V, the SRRs are misaligned (Figure 3d) and Δ≈20 μm.

The EM response of the comb-drive BC-SRR array was characterized using THz time domain spectroscopy (THz-TDS)^36^. The metamaterial was mounted on a sample holder such that the THz pulses were at normal incidence to the sample with the electric field normal to the gaps in the SRRS. A DC voltage supply was utilized to drive the MEMS actuator to control the lateral shift. We measured the static response of the tunable metamaterial by scanning the spectra at a fixed lateral shift. A reference sample, which was an empty silicon frame whose dimension was exactly the same as the metamaterial sample but without SRRs, was measured prior to each scan to provide an accurate reference signal and to eliminate the measurement errors arising from beam clipping by the silicon frame. Details of the THz-TDS setup can be found in the Supplementary Information.

**Figure 4:**
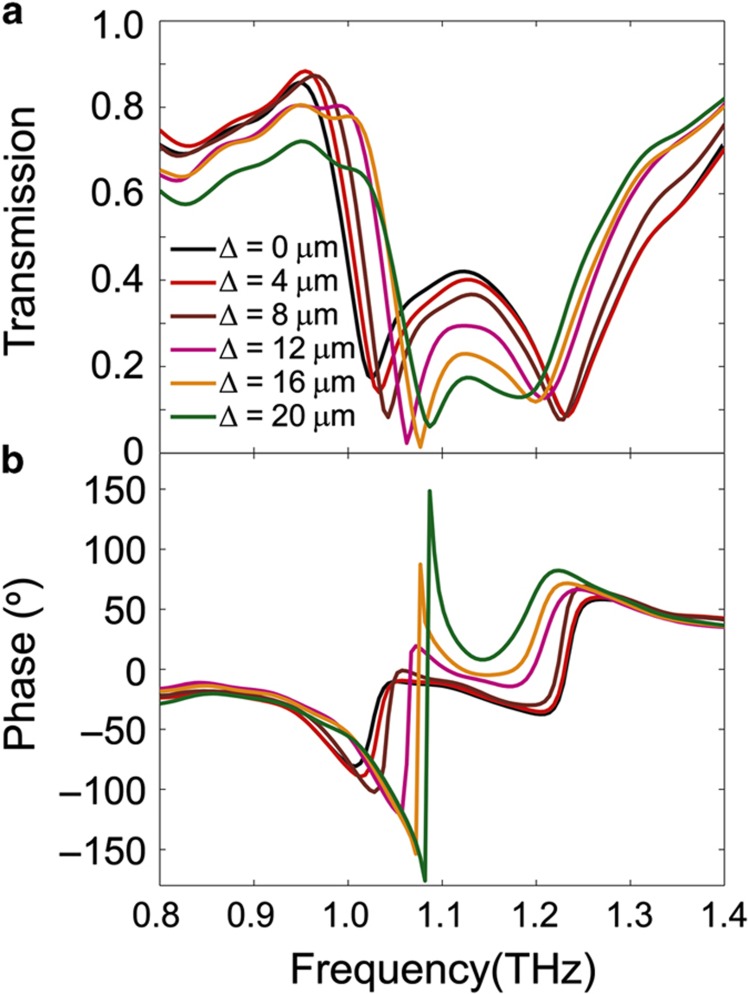
The measured transmission spectrum at different lateral shifts. (**a**) Magnitude and (**b**) phase. From **a**, we can see that two resonant modes exist when the SRRs are aligned (Δ=0 μm). As Δ increases, the first mode shifts to a higher frequency and the second mode shifts to a lower frequency owing to the change of coupling between the SRRs. The phase of the transmission signal is also modulated by the lateral shift as shown in **b**. SRRs, split-ring resonators.

When the SRRs are aligned (Δ=0 μm), as shown by the black curve in Figure 4a, two resonance dips are observed in the transmission spectrum at 1.03 and 1.23 THz. They correspond to the two resonant modes described above. With the increasing lateral displacement of the BC-SRRs, the symmetric mode shifts to higher frequencies, and the anti-symmetric mode shifts to lower frequencies, with the decrease of the amplitude at the resonance. In addition to the magnitude, the phase of the transmitted THz wave, as shown in Figure 4b, is modulated with the lateral displacement of the BC-SRRs. The phase is wrapped to be constrained to the range of (−180°, 180°], so there are sudden phase jumps around the first resonant frequency for Δ=16 and 20 μm in Figure 4b. Clearly, the actuation of the lateral displacement tunes the magnitude and phase of the transmission of our metamaterial device.

To explain the experimental results and understand the tuning mechanism, we use a combination of the equivalent circuit model and finite element analysis with a commercial numerical EM simulation tool (CST Microwave Studio) to study the behavior of the BC-SRRs. The geometries of the SRRs in the simulation are obtained by measuring the device dimensions. The excitation is applied by a wave-guide port with the electric field perpendicular to the SRRs’ gaps. The lateral shift is swept from 0 to 20 μm. The electric conductivity of gold is chosen to be 4.5×10^7^ S m^−1^. The dielectric constants of the silicon substrate and SiN_x_ film are 11.9 and 7.0, respectively. The frequency solver is employed to simultaneously yield the transmission spectra and spatial distributions of the surface current and electric field, which can be used to find the charge distribution.

The BC-SRRs can be considered as two RLC resonators with mutual inductance and capacitance. Two resonant modes can be observed owing to mode splitting. The resonant frequency of each mode can be estimated by

$$f_{r,i}=\frac{1}{2\pi \sqrt{L_{total,i}\cdot C_{total,i}}},i=1,2$$

where *L*_total,*i*_ is the total inductance and *C*_total*,i*_ is the total capacitance of each mode, with *i*=1, 2 corresponding to the symmetric and anti-symmetric modes, respectively. The total inductance is determined by the self-inductance of the two resonators (*L*_self_) and the mutual inductance (*L*_mutual,*i*_) by *L*_total,*i*_=*L*_self_+*L*_mutual,*i*_. Similarly, the total capacitance is *C*_total,*i*_=*C*_self_+*C*_mutual,*i*_.

**Figure 5:**
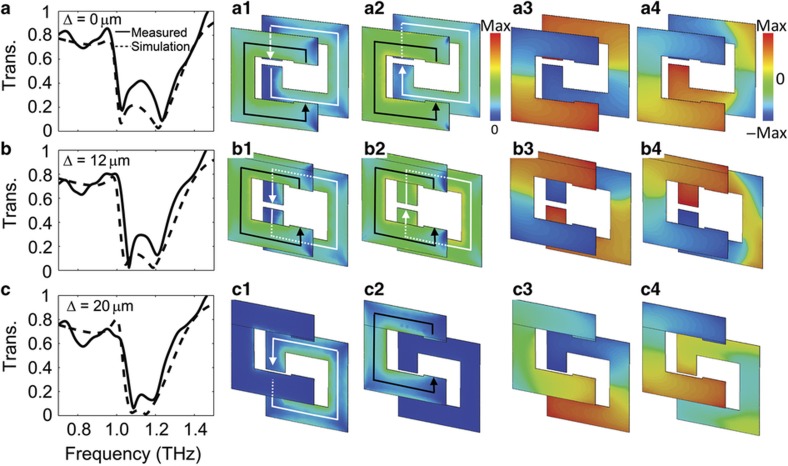
(**a**, **b**, and **c**) Measured and simulated transmission spectra at different lateral shifts. The solid lines are the measured spectra, and the dash lines are the simulation spectra. The second and third columns are the simulated surface current distributions for the 1st and 2nd resonance modes, where the lines with arrows represent the direction of the currents. The fourth and fifth columns are the simulated charge distributions for the 1st and 2nd modes, where red represents positive charge and blue represents negative charge.

Figure 5a presents experimental and simulation transmission spectra of the aligned SRRs, that is, Δ=0 μm. There are two resonant modes (1.03 and 1.23 THz, respectively) in the measured spectrum owing to frequency splitting. The incident THz pulses excite surface currents in the SRRs, leading to electric and magnetic dipoles in the metamaterial^35^. The dipoles radiate to the far field. On resonance, that is, 1.03 THz and 1.23 THz, the dipolar radiation destructively interferes with the incident electromagnetic wave, resulting in near zero transmission. Away from resonance, the transmission is near unity owing to the absence of destructive interference^37^. The surface current simulation results Figures 5a1 and a2 show that the first mode corresponds to a symmetric current distribution and the second one to an anti-symmetric current distribution. Thus, the magnetic fields induced by the SRRs are in the same direction at the first mode and in the opposite direction at the second mode. This indicates that *L*_mutual,1_>0 and *L*_mutual,2_<0. From the numerical simulation, we can retrieve the charge distribution in the SRRs, as shown in Figures 5a3 and a4. For the symmetric mode, the positive and negative charges overlap between the coupled SRRs, resulting in a positive mutual capacitance (*C*_mutual,1_>0). For the anti-symmetric mode, there are charges with identical polarities in the overlapped area of the two SRRs, which decreases the total capacitance. Hence, a negative mutual capacitance between the coupled SRRs exists in this mode (*C*_mutual,2_<0). This means that *L*_total,1_>*L*_total,2_ and *C*_total,1_>*C*_total,2_; as a result, the symmetric mode resonates (as expected) at a lower frequency than the anti-symmetric mode.

With increasing Δ, the magnetic interaction between the BC-SRRs weakens, leading to decreased *L*_mutual*,*1_ and increased *L*_mutual*,*2_. For the electric interaction, the overlapping area of the opposite charges is decreased for the symmetric mode but increased for the anti-symmetric mode, that is, *C*_mutual*,*1_ decreases and *C*_mutual*,*2_ increases. The changes in the mutual inductance and capacitance lead to frequency shifting. An increased Δ will shift the symmetric mode to a higher frequency and the anti-symmetric mode to a lower frequency. For example, when Δ increases from 0 to 12 μm, the symmetric mode shifts from 1.02 to 1.06 THz, and the anti-symmetric mode shifts from 1.21 to 1.20 THz in the experimental result. This agrees well with both the simulation and the qualitative analysis, as shown in Figure 5b.

When the actuator approaches the limit of its travel distance (Δ=20 μm), the SRRs in each pair are misaligned, with the relative positions shown in Figure 3d. Figure 5c shows the corresponding experimental and numerically simulated spectra. In this condition, the induced magnetic field in the center of one resonator passes through the metal edge of the other one such that the magnetic interaction is small (*L*_mutual*,i*_≈0). Meanwhile, the charges on one ring are nearly equidistant from both negative and positive charges on the other ring. Thus, the mutual capacitance is small (*C*_mutual*,i*_≈0), and there is little coupling between the resonators. The simulated surface current and charge distribution results (Figures 5c1–c4) illustrate that the resonance in each SRR is decoupled. The first resonant frequency corresponds to the LC mode of SRR2, and the second corresponds to SRR1. The geometries of the SRRs (Figure 1c) are optimized to match the resonant frequencies of the two uncoupled SRRs in each pair. However, owing to the imperfections in the fabrication, the resonant frequencies of the two SRRs are not perfectly matched in the experimental results. In the simulation, we account for the frequency mismatch and achieve good agreement with the experimental results.

In the experimental and simulation results (Figure 4a and Figures 5a–c), it should be noticed that the amplitude of the transmission at the resonant frequency decreases with increasing lateral shift. This originates from a decreased coupling between the SRRs. The resonance transmission amplitude of an SRR-based metamaterial is related to the strength of excited dipoles, which depends linearly on the induced current in the SRRs^38^. Stronger dipoles will lead to weaker transmission and stronger reflection at the resonant frequency^18^. The BC-SRRs can be considered two inductively coupled second-order resonators (neglecting capacitive coupling for simplification). The amplitude of the induced current in each SRR will increase with a decrease in the coupling strength, thereby leading to a decrease in the transmission amplitude at the resonant frequency (further details are provided in the Supplementary Information).

**Figure 6:**
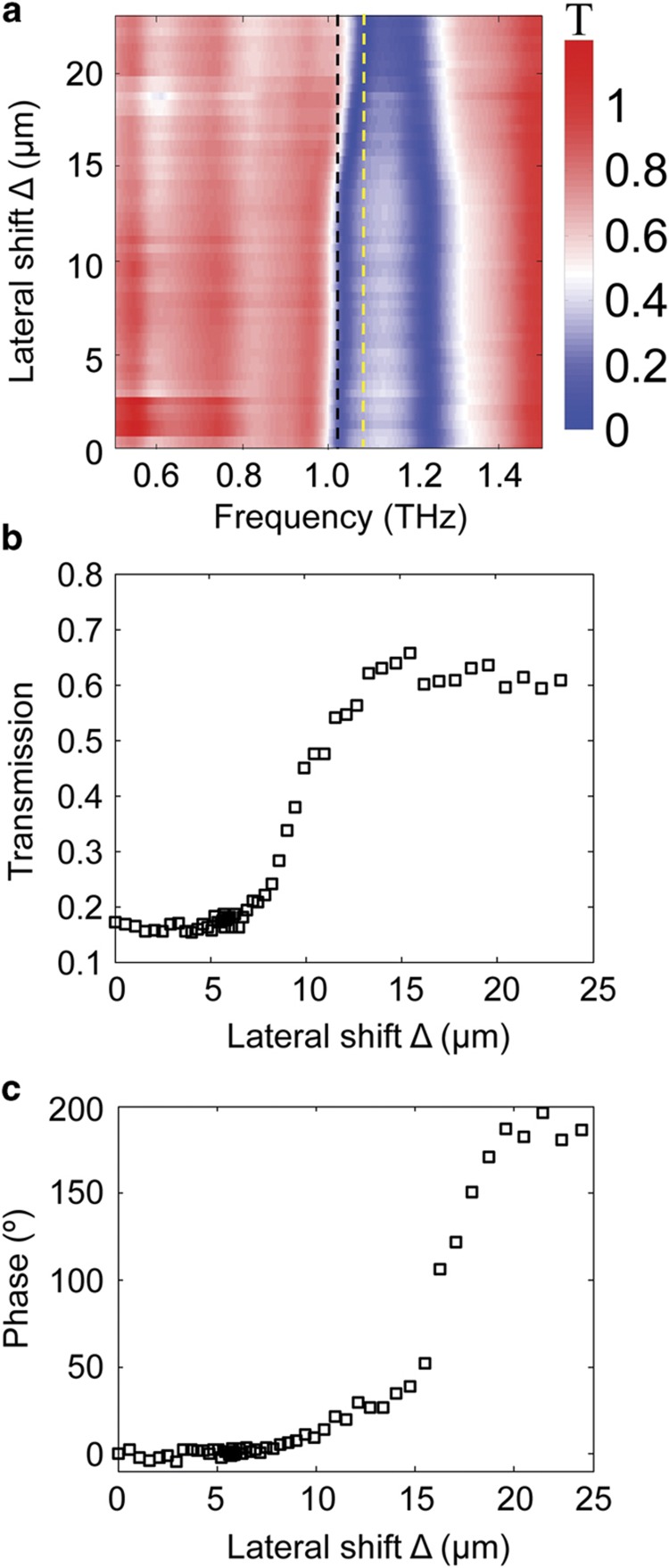
(**a**) Experimental dynamic tuning of the transmission spectrum represented by contour maps of transmission, where the *x* axis is frequency, the *y* axis is the lateral shift, and color represents the magnitude of transmission. (**b**) Magnitude modulation of transmission at 1.03 THz (along the black dash line in **a**). (**c**) Phase modulation of transmission at 1.08 THz (along the yellow dash line in **a**).

By sweeping the lateral shift with a 2 V incremental step in the applied voltage, the contour map of the Δ-dependent THz transmission magnitude is measured, as shown in Figure 6a. When Δ is <10 μm, very little shift in the resonant frequency is observed. When Δ is >10 μm, the resonance shift becomes significant. From the contour map, we can obtain the dynamic magnitude tuning range for a specific frequency. For example, the magnitude of transmission increases from 16 to 63% when the lateral shift is tuned from 0 to 20 μm at 1.03 THz, as shown in Figure 6b. It is not linearly dependent on the lateral shift and increases steeply when 8 μm<Δ<15 μm. The phase tuning range can be obtained as well. Figure 6c illustrates that the phase can be modulated from 0 to 180° at 1.08 THz. Thus, we demonstrate that the magnitude and phase of the transmission signal can be tuned in real-time by choosing the appropriate lateral displacement between BC-SRRs.

On the basis of the structural parameters, the comb-drive actuator can be considered a second-order system with a natural frequency of 760 Hz. This means that we can drive the movable frame to the expected position with an applied voltage in <1.3 ms under critically damped conditions. If a higher modulation speed is required, then we can improve the response time with the help of vacuum packaging and closed loop feedback control.

## Discussion

From the experimental and simulation results, we demonstrate the dynamic tuning of the metamaterial transmission response by driving the vertical lateral shift with a MEMS actuator. In a pair of BC-SRRs, the coupling also depends on the lateral shift along the *x* axis (horizontal lateral shift) in Figure 3b. The misalignment along the *x* axis may decrease the coupling strength between the SRRs, thereby reducing the resonance tuning range. The alignment accuracy along the *x* axis should be ensured to keep a high resonance tuning range for our current design. In contrast, the horizontal lateral shift dependency of the coupling can provide us with an additional modulation of the metamaterial response. If we mount the movable SRRs on a two-dimensional actuator that can move both vertically and horizontally, a larger tuning range can be achieved.

In our current design, resonant frequency matching of the non-interacting resonators helps maximize the tunability upon coupling. From the simulations, we compared the tuning of the response under different frequency mismatches that were achieved by changing the dimensions of the capacitive gaps (that is, *g*_1_ and *g*_2_ in Figure 1a). We find that the frequency tuning range and amplitude modulation depth decrease with the increase of the frequency mismatch (additional details are in the Supplementary Information). Because the tunable response of the BC-SRRs is based on inductive and capacitive coupling, we can modulate the response with the lateral shift, even though there is a large resonant frequency mismatch between the resonators. In an extreme case, a tunable amplitude response can be achieved with an SRR and a non-resonance closed ring^39^. However, if we require both large frequency tuning and amplitude tuning, then resonant frequency matching should be achieved.

According to our simulations, the periodicity of the BC-SRRs has little effect on the resonant frequencies when the SRRs are aligned (Δ=0 μm), as shown in Supplementary Figure S5a in the Supplementary Information. However, it can affect the quality factor of each mode. A larger periodicity leads to a higher quality factor for each mode owing to decreased radiation loss^40^. The tunability of the resonant frequency is not significantly affected by the periodicity. It is possible to optimize the quality factor for different applications by choosing the appropriate periodicity (additional details are in the Supplementary Information).

Moreover, the out-of-plane separation distance between the BC-SRRs also affects their coupling. A smaller separation can lead to stronger coupling between the SRRs, and a larger tuning range can be achieved. According to our experiments, if the separation distance is too small (below ~10 μm), then the movable silicon frame in Chip 1 tends to stick to the fixed SiN_x_ thin-film on Chip 2, leading to the failure of the comb-drive actuators. Thus, the yield of the tunable metamaterial is low, and a larger separation distance is preferred to ensure the stability and yield. A trade-off between the tuning range and yield is achieved at an out-of-plane separation distance of 20 μm by controlling the thickness of the polyimide bonding pads and flip chip bonding parameters. Further optimization of the actuator structure design and fabrication process is required to minimize the separation distance and enhance the tuning range of the metamaterial.

In addition to the out-of-plane separation distance, the thickness of the device layer in the SOI wafer has an important role in determining the stability of the dual-layer metamaterial. A thicker device layer provides stiffer support along the out-of-plane direction, thereby enhancing the device stability. However, the increase in thickness would also detune the SRRs in the two layers and result in a lower quality factor of the device owing to the increased THz loss (based on our simulations). Furthermore, the Fabry–Perot reflection would arise when the device layer has a large thickness. In the present work, we make a trade-off by choosing a 10-μm-thick device layer. In the future, we can optimize the device layer thickness by balancing the mechanical stiffness and THz performance according to the application requirement. The geometry of the SRRs should be redesigned to match their resonant frequencies for the chosen device layer thickness.

The tunable THz metamaterial based on BC-SRRs described in this paper has potential applications in spatial light modulation, THz sensing, and tunable filters. Notably, one of the most appealing applications of this technology is chemical sensing. Many chemicals exhibit unique THz spectral fingerprints, such as explosives^41^, pharmaceuticals,^42^ and biomolecules^43^. THz metamaterials may increase the sensitivity, thereby enabling the detection of trace-amounts of analyte via mode coupling between the metamaterial and analyte of interest^8^. However, the narrow bandwidth of the metamaterials limits their selectivity and causes false positives. Using our tunable metamaterial, we can sweep the resonant frequency dynamically to make it couple with the multiple vibrational modes of the analyte in the THz range to reduce such limitations and improve the selectivity and accuracy. In addition, we have provided a robust and versatile platform for dynamic broadside-coupling studies. One can mount different resonator structures, rather than SSRs, on the two layers to realize diverse functions. For instance, one layer of the metamaterials could be a closed ring array^39^ to allow for dynamic manipulation of the metamaterial oscillator strength and electric field enhancement factor, with the lateral shift controlled by the comb-drive actuator. Moreover, the dimension of the resonators can be scaled to construct devices working at other frequency regimes.

In conclusion, this paper presents the design, fabrication, and characterization of a MEMS-enabled tunable THz metamaterial based on broadside-coupled SSRs, which can modulate both the magnitude and phase of the transmitted EM wave through tuning of the resonant modes. In this design, the symmetric mode blueshifts from 1.02 to 1.08 THz, and the anti-symmetric mode redshifts from 1.23 to 1.18 THz with a 20-μm modulation of the lateral displacement. The mechanism of the tuning is studied qualitatively with an intuitive circuit model by comparing the experimental and simulation results. This tuning mechanism can be applied to other BC-SRR designs working at other designated frequencies.
