# Supplementary material for: Voltage-tunable dual-layer terahertz metamaterials
Source: Microsyst Nanoeng. 2016 Jul 4;2:16025. doi: 10.1038/micronano.2016.25 (PMC6444717; doi:10.1038/micronano.2016.25)
Supplement: Supplementary Information [file micronano201625-s1.pdf]

Supplementary file

Voltage-tunable dual-layer terahertz metamaterials

Xiaoguang Zhao[1], Kebin Fan[1,*], Jingdi Zhang[2,3], George R. Keiser[3,†], Guangwu Duan[1], Richard D. Averitt[2,3] and Xin Zhang[1]

Microsystems & Nanoengineering (2016) **2,** 16025; doi:10.1038/micronano.2016.25; Published online: 4 July 2016

FABRICATION OF THE TUNABLE METAMATERIAL

The tunable metamaterial is fabricated with bulk micromachining processes as shown in Figure S1, which includes two wafer processes and flip chip bonding. The first wafer is an silicon-on-insulator (SOI) wafer with a 10-μm-thick device layer and 2 μm buried oxide layer. Both sides of the wafer are coated with 400-nm-thick low-stress SiN_x films grown by LPCVD. The SiN_x film on the top side is patterned with photolithography and reactive ion etching (RIE) (Figure S1a). Subsequently, the electrodes are deposited on the exposed silicon using e-beam evaporation of 10-nm thick Cr and 400-nm Al followed by a lift-off process. Annealing at 450 °C in H_2/N_2 ambient is undertaken in order to form the ohmic contact. To achieve good contact for the wire bonding, the electrodes are covered with a 150-nm gold thin film after annealing (Figure S1b). Subsequently, 150-nm thick gold SRRs are patterned via a lift-off process (Figure S1c) and the device layer is etched using deep reactive ion etching (DRIE) to form the electro-mechanical structures including the comb-drive actuator and supporting beams (Figure S1d). In order to protect the patterned device layer from damage in the following steps, a 600-nm-thick SiO_2 passivation layer is deposited on the front side using PECVD (Figure S1e). Then, backside alignment and photolithography is performed and the SiN_x film is etched with RIE, followed by etching through the Si substrate using DRIE (Figures S1f and g). The last step (Figure S1h) is to etch the buried oxide layer and passivation layer with Silox Vapoxy III etchant (Transene, Inc., Danvers, MA, United States) to release the movable structure. The second wafer, which is a single crystal $<100>$ silicon wafer, is coated with low-stress SiN_x film on both sides. Subsequently, 150-nm-thick gold SRRs are patterned on the top side with lift-off process (Figure S1i). Then, the silicon wafer is etched through with KOH wet etching from the window opened on the backside SiN_x film (Figure S1j). After the wafer-level fabrication, the two wafers are diced into chips. For clarity and conciseness, we name the chips with comb-drive structures on SOI substrate as Chip 1 and the others as Chip 2. Polyimide bonding pads are patterned on Chip 2 via photolithography of the

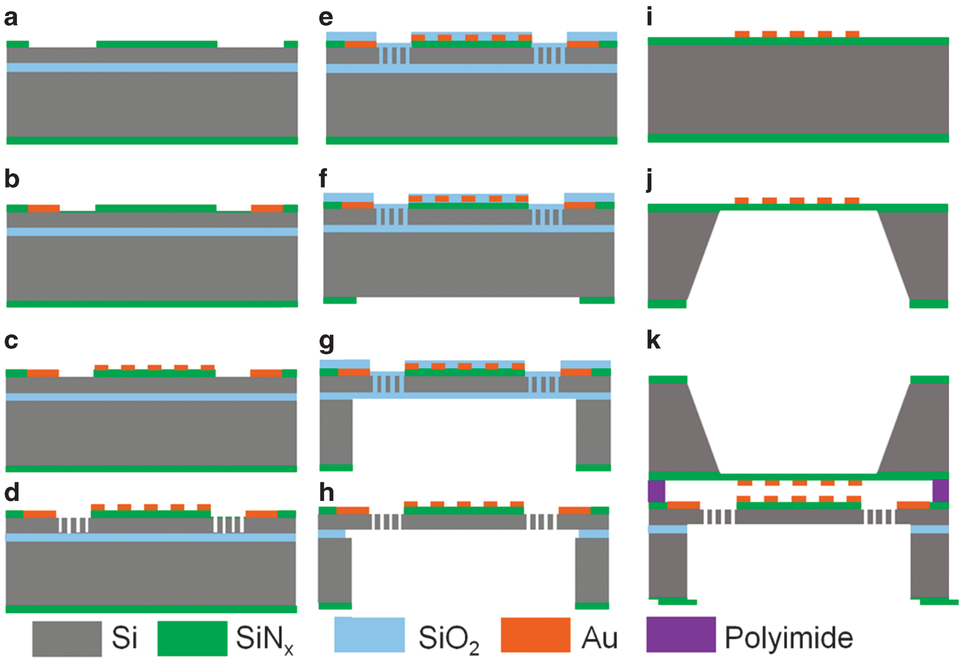

Figure S1 Fabrication process flow of the tunable metamaterial.

[1]Department of Mechanical Engineering, Boston University, Boston, MA 02215, USA; [2]Department of Physics, University of California, San Diego, La Jolla, CA 92093, USA and [3]Department of Physics, Boston University, Boston, MA 02215, USA.
Correspondence: Richard D. Averitt (raveritt@ucsd.edu) or Xin Zhang (xinz@bu.edu)
*Present address: Department of Electrical and Computer Engineering, Duke University, Durham, NC 27708, USA
†Present address: School of Engineering, Brown University, Providence, RI 02912, USA

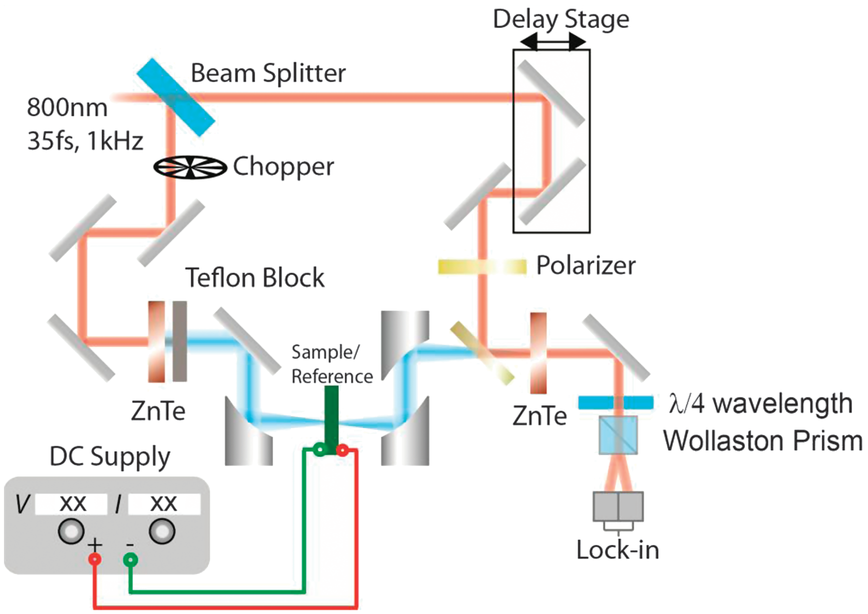

Figure S2 Terahertz time domain spectroscopy for the metamaterial characterization.

photosensitive polyimide (HD8820, HD Microsystems, Parlin, NJ, United States). Finally, we bond a pair of Chips 1 and 2 together with ± 2 μm alignment error using a flip chip bonder (FC150, Suss MicroTec AG, Garching bei München, Germany) under 200 g force and 175 °C temperature.

CHARACTERIZATION OF THE METAMATERIAL

The electromagnetic response of the real-time tunable metamaterial is characterized by THz time domain spectroscopy (THz-TDS), as shown in Figure S2. A 1 kHz Ti:sapphire regenerative amplifier laser producing 1.55 eV near-infrared pulse (800 nm, 3 mJ, 35 fs) was utilized. A Teflon plate blocks the residual incident 800 nm optical beam and allows the THz beam to pass. The THz pulses are focused on the sample or the reference with a plane mirror and a parabolic mirror. The electric field of the THz pulses is polarized perpendicular with the gaps of the SRRs. The transmitted THz pulses are collimated and focused in the detection ZnTe crystal with a pair of parabolic mirrors. The 800-nm optical detection pulses are also fed to the ZnTe crystal. The THz field rotates the polarization of the detection pulses due to the Pockels effect. The polarization rotation is proportional to the electric field strength of the THz pulses. Then, the detection pulses are fed to a quarter-wave plate, converted to be elliptically polarized, and split into two orthogonal linearly polarized pulses with a Wollaston prism. A pair of balance photodiodes are used to detect the two orthogonal pulses and the differential signal, measured by a lock-in amplifier, is an indicator of the instantaneous THz electric field. The THz electric field is measured in the time domain by controlling the delay time between the THz pulses and the detection pulses with a motorized delay stage. We can obtain the time domain signal of the sample and reference individually. Then, a Fourier transform is performed on the time domain signal to obtain the frequency spectrum of the reference [$E_r(\omega)$] and metamaterial samples ($E_s(\omega)$). The transmission spectrum of the sample is calculated by $T(\omega) = E_s(\omega)/E_r(\omega)$. A DC voltage power supply is used to apply voltage to the comb-drive actuator. By sweeping the applied voltage, the spectra of the metamaterial are measured at different lateral shifts.

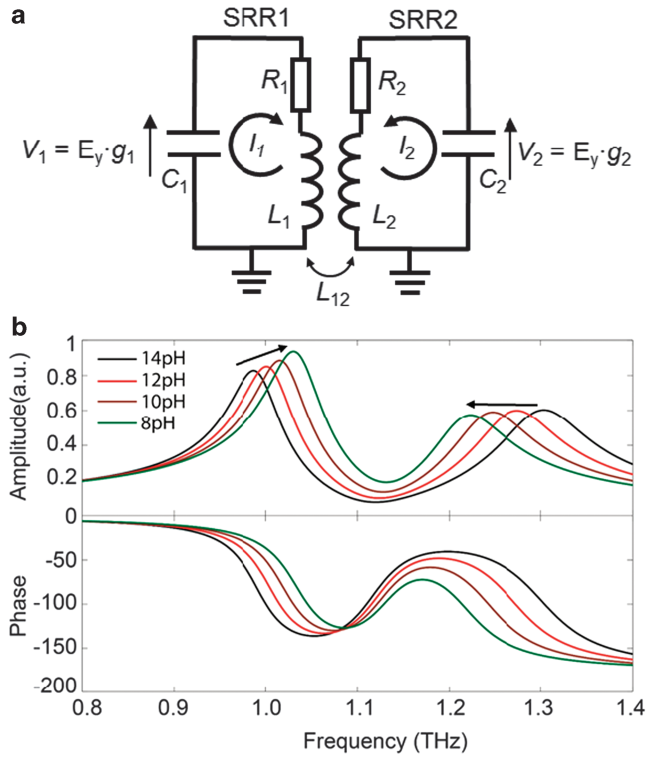

Figure S3 (**a**) Lumped model of the BC-SRRs; (**b**) the electric dipole density frequency response over the incident field strength with different mutual inductances.

SIMPLIFIED LUMPED MODEL OF THE BC-SRRS

The BC-SRRs can be simplified as two RLC resonators with mutual inductance L_{12} (neglecting the mutual capacitance for simplification) as shown in Figure S3a. They are excited by *y*-component of the incident electric field (E_y) as shown in Figure 1b. Using

Kirchhoff's voltage law[1], the current in each SRR can be solved using the following coupled equations:

$$\frac{1}{C_1}\int I_1 dt + R_1 I_1 + L_1\frac{dI_1}{dt} - L_{12}\frac{dI_2}{dt} = E_y g_1$$

$$\frac{1}{C_2}\int I_2 dt + R_2 I_2 + L_2\frac{dI_2}{dt} - L_{12}\frac{dI_1}{dt} = E_y g_2$$

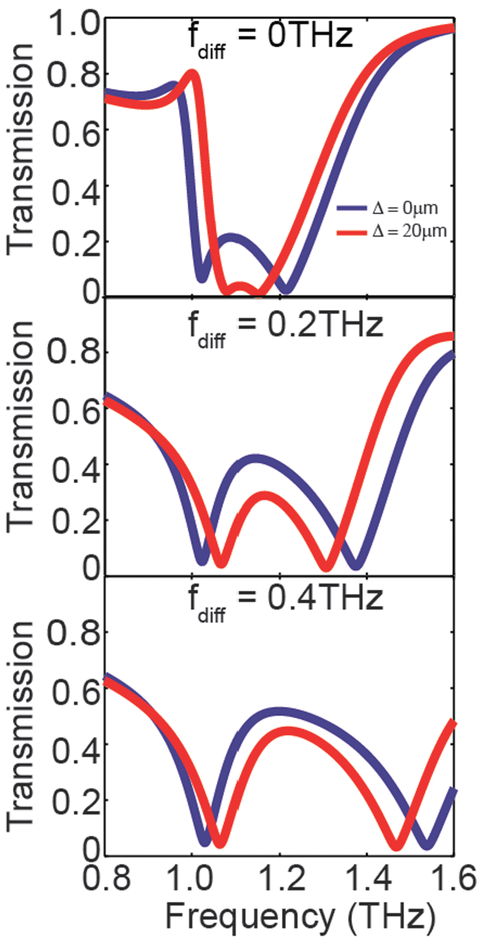

Figure S4 The simulated tunable transmission response of the BC-SRRs with different resonance frequency mismatch (f_{diff}) with 20 μm lateral shift.

where C_i, R_i, and L_i are the lumped capacitance, resistance and inductance of the i th SRR, I_1 and I_2 are the current in SRR1 and SRR2, g_1 and g_2 are the capacitive gap in SRR1 and SRR2, and L_{12} is the mutual inductance between the SRRs.

The transfer function of the system can be determined by solving the equations

$$\frac{1}{C_1 s} I_1(s) + R_1 I_1(s) + L_1 s I_1(s) - L_{12} I_2(s) = E_y(s) g_1$$

$$\frac{1}{C_2 s} I_2(s) + R_2 I_2(s) + L_2 s I_2(s) - L_{12} I_1(s) = E_y(s) g_2$$

The values of C_i, R_i, and L_i can be extracted from the numerical simulation with CST Microwave Studio and are listed in Table S1. We can calculate $I_1(s)/E_y(s)$ and $I_2(s)/E_y(s)$ with these parameters.

According to Ref. 1, the total electric dipole density in the BC-SRRs unit-cell is given by

$$P_y(t) = \frac{1}{V}\left(g_1 \int I_1 dt + g_2 \int I_2 dt\right)$$

where V is the volume of each unit-cell calculated by $V = P^2(d+t)$, in which P is the periodicity of the SRRs, d is the vertical distance between the SRRs, and t is the thickness of the silicon frame in SRR1. In the frequency domain, we can get

$$P_y(s) = \frac{1}{Vs}[I_1(s) + I_2(s)]$$

The frequency response of electric dipole density (P_y) is plotted in Figure S3b. We assume that the mutual inductance is 14 pH for the aligned case. There are two peak values in amplitude spectrum of P_y, corresponding to the resonant frequencies of the BC-SRRs. The peak values in the electric dipole density correspond to the transmission minima of the BC-SRRs (black curve in Figure 4a). With increasing lateral shift, the mutual inductance becomes smaller. The first mode shifts to higher frequency and the second

Table S1 The parameters used in the lumped model

Parameters	Value
C_1 (fF)	0.31
R_1 (Ω)	21
L_1 (pH)	62
C_2 (fF)	0.45
R_2 (Ω)	30
L_2 (pH)	47
g_1 (μm)	14
g_2 (μm)	2

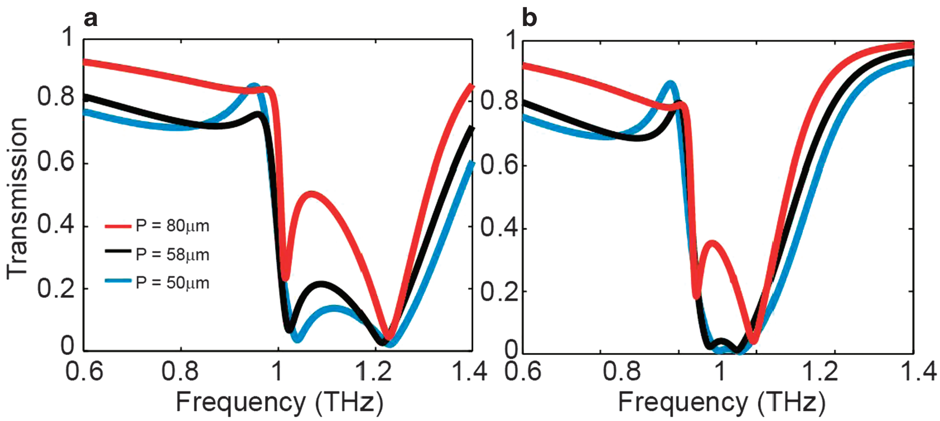

Figure S5 The transmission spectra of BC-SRRs with different periodicities (P) for (**a**) $\Delta = 0$ μm and (**b**) $\Delta = 20$ μm.

Table S2 The tunability of BC-SRRs with different frequency mismatch

Frequency mismatch (f_{diff})	Resonance shift of mode 1	Resonance shift of mode 2	Modulation depth at 1.03 THz
0 THz	60 GHz	60 GHz	0.61
0.2 THz	40 GHz	60 GHz	0.23
0.4 THz	30 GHz	60 GHz	0.18

mode shifts to the lower frequency. At the same time, the peak value of the electric dipole density at the first mode increases, resulting in a decreased transmission at the resonant frequency. However, the electric dipole density at the second mode almost keeps constant. The change of electric dipole density is one reason of the change in the transmission response. Even though we only take the inductive coupling into account (e.g we neglect capacitive coupling and other effects, such as bianisotropy of the BC-SRRs), this simple model can be used to understand the basic response of the coupled SRRs and provide insight into the experimentally observed transmission changes.

THE EFFECT OF RESONANT FREQUENCY MISMATCH

In this paper, a pair of resonance frequency matched SRRs are used in the metamaterial unit-cells. To investigate the effect of frequency mismatch on the tunable response of the metamaterials, the gap of SRR2 (g_2) is changed to introduce resonance frequency mismatch (f_{diff}) between SRR1 and SRR2 in the simulations using CST Microwave Studio. The tunable transmission of the BC-SRRs with different resonance mismatch for 20 μm lateral shift are plotted in Figure S4. The tuned parameters, i.e. resonance frequency shift of Modes 1 and 2 and the modulation depth at 1.03 THz, are listed in Table S2 for a 20 μm lateral shift. From the results, we can conclude that the BC-SRRs with matched resonance frequency have the greatest tunability and largest modulation depth at 1.03 THz.

THE EFFECT OF PERIODICITY

The numerically simulated transmission spectra of the BC-SRRs with different periodicities are shown in Figures S5. When the BC-SRRs are aligned ($\Delta = 0$ μm), the resonance dip of each mode becomes sharper with an increase of the periodicity, indicating an increased quality factor. Resonant frequency tuning of the BC-SRRs is not strongly affected by the periodicity for a constant lateral displacement (i.e. by comparing Figure S5a and b). However, if a larger lateral displacement can be realized by the actuator, larger tuning may be achieved. For our current case, P is 58 μm and L is 40 μm, we cannot misalign the array of SRRs completely because the movable SRRs overlap with the adjacent SRRs when the lateral shift is larger than $P/2$. As a result, we cannot fully decouple the BC-SRRs. If P is 80 μm and L is 40 μm and we can achieve 40 μm lateral displacement, and there will be no overlapped areas between the BC-SRRs leading to minimum coupling. Currently, due to the limitation in the lateral shift (maximum is ~25 μm), the periodicity has little effect on the tunability. However, we can control the quality factor with the periodicity.

COMPETING INTERESTS

The authors declare no conflict of interest.

REFERENCES

1 Kriegler CE, Rill MS, Linden S *et al.* Bianisotropic photonic metamaterials. *IEEE Journal of Selected Topics in Quantum Electronics* 2010; **16**: 367–375.

2 He X. Tunable terahertz graphene metamaterials. *Carbon* 2015; **82**: 229–237.
